# Predicational ability of phase angle on protein energy wasting in kidney disease patients with renal replacement therapy: A cross‐sectional study

**DOI:** 10.1002/fsn3.2310

**Published:** 2021-05-07

**Authors:** Haiteng Zhou, Wenlong Yao, Da Pan, Guiju Sun

**Affiliations:** ^1^ Key Laboratory of Environmental Medicine and Engineering Ministry of Education Department of Nutrition and Food Hygiene School of Public Health Southeast University Nanjing China; ^2^ Jingjiang People’s Hospital Jingjiang China

**Keywords:** body composition, chronic kidney disease, mid‐arm circumference, phase angle, protein energy wasting

## Abstract

**Objective:**

To investigate the ability of phase angle (PA) and body composition for predicting protein energy wasting (PEW) in renal replacement therapy (RRT) patients.

**Methods:**

Renal replacement therapy (RRT) patients were enrolled in this study. Body composition was measured by direct segmental multi‐frequency biolectrical impedance analysis method (DSM‐BIA); phase angle (PA), fat‐free mass (FFM), fat mass (FM), mid‐arm circumference (MAC), WC (waist circumference), and ECW/TBW (extracellular water/total body water) were obtained. Biochemicals (serum albumin, triglyceride, and cholesterol) were tested. PEW patients were classified according to ISRNM (The International Society of Renal Nutrition and Metabolism) criteria. Cutoff value of PA and related variables was calculated by ROC analysis. The ability of body composition variables as indicators to predict PEW was evaluated.

**Results:**

Sixty‐four patients were enrolled in this study. Thirty‐three patients (52.6%) were males, and forty (62.5%) patients were diagnosed with PEW. The ROC curve showed that the optimal cutoff values of PA, FFMI (fat‐free mass index), MAC, WC, and BMI for PEW risk were 4.45°, 16.71, 29.7 cm, 86.4 cm, and 21.1 kg/m^2^, respectively. These indicators showed significant association with PEW; meanwhile, the PA and MAC can be used as the predictors for PEW with OR 6.333 (95% CI, 1.956–20.505) and 3.267 (95% CI, 1.136–9.394), respectively. Both groups have a lower BUN/Cr ratio (<20).

**Conclusion:**

In the RRT patients, over than 60% patients were diagnosed with PEW. PA, MAC, and other body composition can be used as the independent indicators for predicting PEW in renal replacement therapy kidney disease patients.

## Graphical abstract

Protein energy wasting is prevalence in chronic kidney disease (CKD) patients on renal replacement therapy by the International Society of Renal Nutrition and Metabolism. Phase angle and mid‐arm circumference can be used as the independent and reliable indicators for the noninvasive predicting of protein energy wasting and evaluating nutrition status in CKD patients.

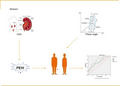

## 1 INTRODUCTION

Chronic kidney disease (CKD) is a common public health disease globally. In South Asian region, the incidence of CKD ranges from 10.2% to 21.2%, which is similar to the global prevalence (13.4%; Hasan et al., 2018; Hill et al., 2016). CKD burden existed in both developing and developed countries. Studies have revealed that nearly 120 million adults were with kidney disease in China (2012), and the prevalence of CKD in USA was 13% in 2007 (Herrera Valdés et al., 2020; Lv & Zhang, 2019). In the past 30 years, the mortality of CKD rised from 20th (1990) to 16th in the leading causes of death (China, 2017; Zhou et al., 2019). The rising number of patients required RRT for it could reduce the complications and kidney burden. CKD also could be affected by factors like diet, physical activity, and metabolic diseases (obesity, diabetes, and hypertension) except for the treatment of RRT (Herrera Valdés et al., 2020; Kelly et al., 2019). In the CKD adverse outcomes, PEW is a denominated problem, and this malnutrition would reduce the patients’ quality of life, would need more healthcare resource and higher medical costs, and would contribute to the mortality risk (Bonanni et al., 2011; Chao et al., 2017). A meta‐analysis showed that the PEW prevalence among 30 countries was 28%–54% (Carrero et al., 2018). In As'habi's cross‐sectional study, the prevalence of PEW in peritoneal dialysis patients was 29% (As'Habi et al., 2019). In Lydia Namuyimbwa's study, PEW prevalence was 47.3% in the CKD subjects and significantly higher than those without CKD (21.3%; Vermeulen, Lopes, Grilo, et al., 2019). Since the dominate prevalence of PEW has great impact on the quality of life and treatments of CKD, the early diagnosis and intervention would benefit the health situation and reduce the medical cost and burden (Chao et al., 2017).

Actually, some biomarkers and indicators could be used for the diagnosis of PEW. ISRNM recommends that serum chemistry, body mass, muscle mass, and dietary intake can be regarded as indicators for PEW diagnosis (Carrero et al., 2013). The criterion is that, if three characteristics are present (low serum levels of albumin (must), reduced body mass and reduced muscle mass), the PEW could be diagnosed. Other biomarkers like C‐reactive protein (hsCRP), log IL‐6, soluble intercellular adhesion molecule‐1 (sICAM‐1), gelsolin, adipokines, serum leptin levels, serum creatinine, and TNF‐alpha were also used to diagnose for PEW (Chiu et al., 2015; Choi et al., 2010). Besides the test indicators, some noninvasive diagnosis also could be used for the screening of PEW. In Arias‐Guillen's study, bioimpedance spectroscopy was used to detect PEW, and results showed that it could be used as a practical instrument to assess nutritional status in patients using body composition (Arias‐Guillén et al., 2018). Srinivasan Beddhu and Castellano‐Gasch also recommended body composition was valid in the diagnosis of PEW (Beddhu et al., 2017; Castellano‐Gasch et al., 2014).

PA was calculated by arctangent (reactance (Xc)/resistance (R)) × (180/π) and could be obtained by bioelectrical impedance analysis in 50 kHz. PA could be used in the evaluation of nutritional assessment, muscle function, and type 2 diabetes (Chen & Zhou, 2019; Dittmar et al., 2015; Player et al., 2019; Yamada et al., 2018). Yoshida's study indicated that patients with severe motor and intellectual disabilities have lower PA value and ECW/TBW (Yoshida et al., 2017). Besides PA, MAC and body composition also could be used as the indicators for PEW diagnosis in RRT patients (Krishnamoorthy et al., 2015; Leal Escobar et al., 2019; Powrózek et al., 2019; Shin et al., 2017). PEW is a malnourished problem and difficult to be assessed, while many studies demonstrated that PA is a practical indicator for this assessment (Player et al., 2019; Tan et al., 2019). Studies on the ability of PA, MAC, and related body compositions in noninvasive diagnosis of PEW are very valuable. This present study aimed to investigate the predicting ability of PA and body composition in the prediction of PEW on RRT patients.

## 2 METHODS AND MATERIALS

A cross‐sectional study was conducted on the patients with the treatment of RRT. Data were collected between January 2018 and June 2019 in Jingjiang People's Hospital, Jiangsu Province, China. This study was a secondary research based on the treatment of RRT patients, and treatment data were collected. Criteria and categories for PEW were reference to the diagnosis criteria of ISRNM, and the diagnosis of PEW was at least 3 of the indicators and one biochemical test presented below: (a) serum levels of albumin less than 3.8 g/dl or total cholesterol <100 mg/dl; (b) BMI less than 23 kg/m^2^; (c) FFMI less than 17.0 kg/m^2^ or 15.0 kg/m^2^ for men and women according to definitions from The European Society of Clinical Nutrition and Metabolism (ESPEN; Cederholm et al., 2015); (d) over‐hydration (ECW/TBW >0.385), which is correlated with inflammation status and subclinical manifestation in CKD patients (Lee et al., 2015; Panorchan & Davenport, 2017; Sasaki & Al, 2008).

### 2.1 Data collection

Direct segmental multi‐frequency biolectrical impedance technology was used to analyze body composition (InBody^®^ model 770). All the patients who finished the hemodiafiltration treatment would be turned to body composition analysis with four couple of electrode holders placed on the ankles and forefingers of the hands, and at least 8 hr or overnight fasting with light clothes on the body. Weight, MAC, WC, fat mass (%), FFM, ECW, and TBW were obtained by this instrument. PA was obtained at a frequency of 50 Hz. Height was measured by height meter (InBody^®^ model BSM 170), and FFMI and BMI were calculated.

### 2.2 Biochemical data

Serum cholesterol, triglyceride, and albumin levels were obtained by medical examination (serum biomarkers must be tested during the hemodiafiltration process), and we conduct a further analysis based on these data.

### 2.3 Statistical analyses

Normal distribution data were presented as mean ± *SD*, and non‐normal numeric variables were presented by median and interquartile distance. Student's *t* test or Fisher's exact test was used to analyze the difference between groups; Spearman's rank correlation was used between body composition and biochemical variables with MAC and PA. Using a receiver operating characteristic curve (ROC) and area under the curve (AUC), cutoff values were calculated. With the ROC optimum cutoff values, chi‐square analysis was conducted, and odds ratio and 95% confidence interval (95% CI) were calculated. *p* value <.05 was considered to be statistically significant.

## 3 RESULTS

Sixty‐four patients were enrolled in this study, thirty‐three (52.6%) were male, and the average age was sixty years old. Forty (62.5%) patients were diagnosed as PEW, and over than 60% patients have edema symptoms. Patient's demographical and body composition parameters are shown in Table 1.

**TABLE 1:** Patient's demographic characteristics

Variables	Mean (percentage)
Men	33 (52.6)
PEW	40 (62.5)
Edema	60 (93.8)
Age	59.6 ± 12.60
Height (cm)	163.6 ± 8.70
Weight (kg)	62.1 ± 12.60
Mid‐arm circumference (cm)	29.2 ± 3.22
Fat‐free mass index (kg/m^2^)	17.3 ± 2.19
Phase angle (°)	4.6 ± 0.78
Body mass index (kg/m^2^)	23.1 ± 3.91
Waist circumference (cm)	81.3 ± 10.94

According to the diagnosis expressed above, patients were divided into PEW and non‐PEW groups. Patients with non‐PEW has a lower weight (*p* = .012). Compared with PEW group, MAC (*p* = .008), cholesterol (*p* < .01), albumin concentration, (*p* = .004), and PA (*p* < .01) were significantly higher in non‐PEW group. Creatinine in serum, BUN in serum, and ECW/TBW showed no statistically significant difference between groups (*p* < .05; Table 2).

**TABLE 2:** Comparison of variables between PEW and non‐PEW CKD patients on hemodiafiltration

Variables	PEW	Non‐PEW	*p* Value
*N*	40	24	—
Age	59.4 ± 12.41	59.9 ± 13.32	.882
Height (cm)	163.3 ± 9.12	164.1 ± 8.15	.709
Weight (kg)	59.1 ± 9.72	67.1 ± 15.23	.012*
Fat‐free mass (kg)	44.9 ± 8.46	49.2 ± 9.47	.070
Body mass index (kg/m^2^)	21.7 ± 2.95	25.5 ± 4.26	.000*
Fat‐free mass index (kg/m^2^)	16.7 ± 1.80	18.1 ± 2.51	.011*
Mid‐arm circumference (cm)	28.4 ± 2.74	30.5 ± 3.57	.008*
Waist circumference (cm)	78.4 ± 8.43	86.1 ± 12.97	.005*
ECW/TBW	0.391 ± 0.01	0.393 ± 0.01	.591
Cholesterol in serum (mg/dl)	65.6 ± 13.70	88.1 ± 29.1	.000*
Albumin in serum (g/dl)	40.4 ± 2.53	43.6 ± 2.69	.004*
Creatinine in serum (µmol/l)	834.8 ± 352.11	804.5 ± 402.54	.754
BUN in serum (mg/dl)	66.2 ± 24.74	67.2 ± 30.34	.890
Phase angle (°)	4.3 ± 0.67	5.0 ± 0.70	.000*

Note

Mean ± *SD*.

* Statistical significance.

Correlations of body composition and biochemical variables with mid‐arm circumference and phase angle were analyzed (Table 3). MAC (*R* = .516, *p* < .01) and WC (*R* = .346, *p* = .005) were positively correlated with PA, while the EW/TBW (*R* = −.5, *p* < .01) was negatively correlated with PA. Phase angle (*R* = .516, *p* < .01), BMI (*R* = .466, *p* < .01), and waist circumference (*R* = .889, *p* < .01) were positively correlated with MAC, while albumin (*R* = −.426, *p* < .01) and ECW/TBW (*R* = −.357, *p* < .004) were negatively correlated with MAC, respectively.

**TABLE 3:** Correlations of body composition and biochemical variables with mid‐arm circumference and phase angle

Variables	Phase angle	
	*R*	*p*
Fat‐free mass(kg)	.086	.498
Albumin in serum (g/dl)	−.096	.452
Mid‐arm circumference(cm)	.516	.000*
BMI (kg/m^2^)	.109	.391
Waist circumference (cm)	.346	.005*
ECW/TBW	−.500	.000*
	**Mid‐arm circumference**	
Fat‐free mass (kg)	.037	.769
Albumin in serum (g/dl)	−.426	.000*
Phase angle (°)	.516	.000*
BMI (kg/m^2^)	.466	.000*
Waist circumference (cm)	.889	.000*
ECW/TBW	−.357	.004*

* Statistical significance.

**FIGURE 1:**
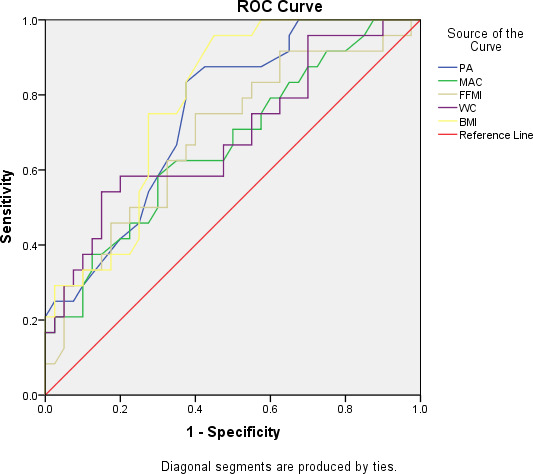
Phase angle, mid‐arm circumference, and other variables as the predictors for protein energy wasting patients

The variables of ROC curve were drawn. AUC, the premium cutoff points, sensitivity, and specificity were calculated. The body composition indicators presented a distinguished diagnosis of PEW on RRT patients (Figure 1). The AUC and premium cutoff points for PA, MAC, FFMI, BMI, and WC were 0.749°, 0.668°, 0.691°, 0.783°, 0.690°, and 4.45°, 29.7 cm, 19.71, 21.1, 86.4 cm, respectively. AUC of PA and BMI was over than 0.7 revealed great ability of the diagnosis of PEW in RRT patients (Table 4).

**TABLE 4:** Variables of AUC and premium cutoff points from ROC

Variables	Area	*p* Value	95% CI	Sensitivity	Specificity
PA <4.45°	0.749	.001	0.630–0.867	83.3	37.5
MAC <29.7 cm	0.668	.026	0.530–0.805	58.3	30.0
FFMI <16.71 kg/m^2^	0.691	.011	0.556–0.827	75.0	40.0
WC <86.4 cm	0.690	.012	0.551–0.828	54.2	15.0
BMI <21.1 kg/m^2^	0.783	.000	0.673–0.893	95.8	45.0

Based on the ROC cutoff points, chi‐square test was conducted between PEW and non‐PEW groups, and OR (odds ratio) and 95% CI (95% confidence interval) were calculated. The cutoff points of PA <4.45° (OR = 6.333, *p* = .002), MAC <29.7 cm (OR = 3.267, *p* = .036), BMI <21.1 kg/m^2^ (OR = 28.111, *p* < .01), and WC <86.4 cm (OR = 7.933, *p* = .001) were identified as the high risk for PEW (Table 5).

**TABLE 5:** Premium cutoff value of phase angle and other variables to predict PEW

Variables	OR	*p* Value	95% CI
PA <4.45^0^	6.333	.002	1.956–20.505
MAC <29.7 cm	3.267	.036	1.136–9.394
BMI <21.1 kg/m^2^	28.111	.000	3.453–228.823
WC <86.4 cm	7.933	.001	2.418–26.030
FFMI <16.71 kg/m^2^	1.629	.562	0.493–5.380

## 4 DISCUSSION

Bioelectrical impedance analysis is a simple, noninvasive, and reliable technique for estimation of body composition and has been used in the diagnosis of diseases such as Type 2 diabetes, muscle dysfunction, hydration, and nutritional assessment (Dittmar et al., 2015; Norman et al., 2012; Vermeulen, Lopes, Grilo et al., 2019). In CKD patients, malnutrition has been widely recognized and the manifested is PEW. Detecting and managing nutrition status would be beneficial to the treatment of patients and decrease the mortality (Bataille et al., 2019; Bolasco et al., 2019). BUN and creatinine are related with the function of renal and conditionally nutrition status. In our study, BUN/Cr was below 20, and there was no difference between PEW and non‐PEW groups. Since creatinine is affected by dietary protein intakes, this result may be inaccurate (Singh et al., 2015). Preview studies have reported that aging was associated with the prevalence of CKD (56.4 years old in Henan China Duan et al., 2019 and 57.2 years old in north Sri Lanka Ranasinghe et al., 2019), and this aging trend was also recognized in our study (the average age was 59.6 years old). Forty (62.5%) patients were diagnosed with PEW in the present study, which was higher than similar studies for the influence of aging (Bi et al., 2019; Gracia‐Iguacel et al., 2019; Hara et al., 2018). Many indicators could be used for determining PEW, such as the ISRNM and ESPEN definitions we refer to. Besides dietary assessment, PEW score, geriatric nutritional risk index biomarkers, and body composition indexes were also reported in the diagnosis of PEW (Ishii et al., 2017; Lee et al., 2019; Monzani et al., 2018). In present study, we evaluated the capability of body composition, especially PA and MAC, in the diagnosis of PEW on CKD patients.

According to ROC analysis, we recommend that patients with PA less than 4.46 degree would have a higher risk of PEW (AUC = 0.749, sensitivity + specificity = 120.8%). The premium cutoff value was similar with Jung‐ho Shin's study (PA <4.5°; Shin et al., 2017). We also analyzed the capability of MAC, WC, BMI, and FFMI in the diagnosis of PEW and recommended that the premium cutoff value was 29.7 cm, 86.4 cm, 21.1 kg/m^2^, and 16.71 kg/m^2^, respectively. AUC demonstrated BMI has better capability for the diagnosis of PEW which was a recommended indicator from ISRNM, ESPEN, and SGA (Vermeulen, Lopes, Alves, et al., 2019) (subjective global assessment). BMI cutoff point in this study was 21.1 kg/m^2^, and Windahl found that PEW was the most common in patients with BMI <22 kg/m^2^ (Windahl et al., 2018). The correlation between body composition and biomarkers was conducted. MAC (*R* = .516, *p* < .01) and WC (*R* = .346, *p* = .005) were positively correlated with PA, while the EW/TBW (*R* = −.5, *p* < .01) was negatively correlated with PA, which was associated with the severity of nutritional status, and consistent result was obtained in Yoshida, M’s study. Phase angle (*R* = .516, *p* < .01), BMI (*R* = .466, *p* < .01), and waist circumference (*R* = .889, *p* < .01) were positively correlated with MAC, while albumin (*R* = −.426, *p* < .01) and EW/TBW (*R* = −0.357, *p* < .004) were negatively correlated with MAC, respectively (Krishnamoorthy et al., 2015; Tan et al., 2019). Considering M‐BIA as predictor in the evaluation of health in populations (Norman et al., 2012; Powrózek et al., 2019; Vermeulen, Lopes, Alves et al., 2019), our result also indicated that PA can be used as independent factor associated with malnutrition in CKD patients.

Karavetian et al. (2019) according to the ROC cutoff points, Chi‐square test was conducted between PEW and non‐PEW groups. OR and 95% CI were calculated in prediction of PEW. PA under 4.46 degree was associated with the prevalence of PEW (OR = 6.333, 95% CI 1.950–20.505). Tan et al. (2019) Identically, MAC <29.7 CM (OR = 3.267, 95% CI 1.136–9.394), BMI <21.1 kg/m^2^ (OR = 28.111, 95% CI 3.418–228.823), and WC <86.4 cm (OR = 7.933, 95% CI 2.418–26.030) showed a higher risk for the incidence of PEW.

Limitations should be notified in this study: firstly, our research objects recruited CKD patients on RRT, and average age was close to 60 years old; thus, results cannot be extrapolated to other patients in different treatment and other age groups; secondly, sample size is limited due to the patients we recruited were only from one hospital within established time.

## 5 CONCLUSION

In the aging RRT patients, more than 60% patients were diagnosed with PEW. PA and MAC can be used as the independent and reliable indicators for the noninvasive prediction of PEW and evaluation of nutritional status in aging CKD patients on RRT.

## CONFLICT OF INTEREST

The authors declared that they do not have any commercial or associative interest that represents a conflict of interest in connection with the work submitted.

## ETHICAL APPROVAL

This study does not involve any human or animal testing.

## INFORMED CONSENT

Written informed consent was obtained from all study participants.

## References

[fsn32310-bib-0001] Arias‐Guillén, M. , Perez, E. , Herrera, P. , Romano, B. , Ojeda, R. , Vera, M. , Ríos, J. , Fontseré, N. , & Maduell, F. (2018). Bioimpedance spectroscopy as a practical tool for the early detection and prevention of protein‐energy wasting in hemodialysis patients. Journal of Renal Nutrition, 28(5), 324–332. 29691162 10.1053/j.jrn.2018.02.004

[fsn32310-bib-0002] As'Habi, A. , Najafi, I. , Tabibi, H. , & Hedayati, M. (2019). Prevalence of protein‐energy wasting and its association with cardiovascular disease risk factors in iranian peritoneal dialysis patients. Iranian Journal of Kidney Diseases, 13(1), 48–55. 30851719

[fsn32310-bib-0003] Bataille, S. , Bon, J. , Kolko, A. , Chauveau, P. , Cluze, J. , Orthwein‐Finck, M. , Mouelhi, Y. , & Mira, M. (2019). Daily practices of protein energy wasting management in hemodialysis patients: A French national survey. Nephrologie & Therapeutique, 15(3), 136–142. 31053553 10.1016/j.nephro.2018.11.008

[fsn32310-bib-0004] Beddhu, S. , Chen, X. , Wei, G. , Raj, D. , Raphael, K. L. , Boucher, R. , Chonchol, M. B. , Murtaugh, M. A. , & Greene, T. (2017). Associations of protein‐energy wasting syndrome criteria with body composition and mortality in the general and moderate chronic kidney disease populations in the United States. Kidney International Reports, 2(3), 390–399. 28840197 10.1016/j.ekir.2017.01.002 PMC5563827

[fsn32310-bib-0005] Bi, X. , Chu, M. , Ai, H. , Hu, C. , & Ding, W. (2019). Association of serum IL‐18 with protein‐energy wasting in end‐stage renal disease patients on haemodialysis. International Urology and Nephrology, 51(7), 1271–1278. 31119516 10.1007/s11255-019-02167-5

[fsn32310-bib-0006] Bolasco, P. , Aquilani, R. , & Murtas, S. R. (2019). Amino acid profile after oral nutritional supplementation in hemodialysis patients with protein‐energy wasting. Nutrition, 62, 211–212. 10.1016/j.nut.2018.11.024 30935712

[fsn32310-bib-0007] Bonanni, A. , Mannucci, I. , Verzola, D. , Sofia, A. , Saffioti, S. , Gianetta, E. , & Garibotto, G. (2011). Protein‐energy wasting and mortality in chronic kidney disease. International Journal of Environmental Research and Public Health, 8(5), 1631–1654. 21655142 10.3390/ijerph8051631 PMC3108132

[fsn32310-bib-0008] Carrero, J. J. , Stenvinkel, P. , Cuppari, L. , Ikizler, T. A. , Kalantar‐Zadeh, K. , Kaysen, G. , Mitch, W. E. , Price, S. R. , Wanner, C. , Wang, A. Y. M. , ter Wee, P. , & Franch, H. A. (2013). Etiology of the protein‐energy wasting syndrome in chronic kidney disease: A consensus statement from the International Society of Renal Nutrition and Metabolism (ISRNM). Journal of Renal Nutrition, 23(2), 77–90. 23428357 10.1053/j.jrn.2013.01.001

[fsn32310-bib-0009] Carrero, J. J. , Thomas, F. , Nagy, K. , Arogundade, F. , Avesani, C. M. , Chan, M. , Chmielewski, M. , Cordeiro, A. C. , Espinosa‐Cuevas, A. , Fiaccadori, E. , Guebre‐Egziabher, F. , Hand, R. K. , Hung, A. M. , Ikizler, T. A. , Johansson, L. R. , Kalantar‐Zadeh, K. , Karupaiah, T. , Lindholm, B. , Marckmann, P. , … Kovesdy, C. P. (2018). Global prevalence of protein‐energy wasting in kidney disease: A meta‐analysis of contemporary observational studies from the international society of renal nutrition and metabolism. Journal of Renal Nutrition, 28(6), 380–392. 30348259 10.1053/j.jrn.2018.08.006

[fsn32310-bib-0010] Castellano‐Gasch, S. , Palomares‐Sancho, I. , Molina‐Núñez, M. , Ramos‐Sánchez, R. , Merello‐Godino, J. I. , & Maduell, F. (2014). New reliable methods for the diagnose of protein‐energy wasting in hemodialysis patients. Nutricion Hospitalaria, 30(4), 905–910. 25335680 10.3305/nh.2014.30.4.7730

[fsn32310-bib-0011] Cederholm, T. , Bosaeus, I. , Barazzoni, R. , Bauer, J. , Van Gossum, A. , Klek, S. , Muscaritoli, M. , Nyulasi, I. , Ockenga, J. , Schneider, S. M. , de van der Schueren, M. , & Singer, P. (2015). Diagnostic criteria for malnutrition – an ESPEN consensus statement. Clinical Nutrition, 34(3), 335–340. 25799486 10.1016/j.clnu.2015.03.001

[fsn32310-bib-0012] Chao, C.‐T. , Tang, C.‐H. , Cheng, R.‐Y. , Wang, M.‐H. , & Hung, K.‐Y. (2017). Protein‐energy wasting significantly increases healthcare utilization and costs among patients with chronic kidney disease: A propensity‐score matched cohort study. Current Medical Research and Opinion, 33(9), 1705–1713. 28699849 10.1080/03007995.2017.1354823

[fsn32310-bib-0013] Chen, W. , & Zhou, S. (2019). Standardized phase angle as a prognostic and nutritional status tool for pancreatic cancer patients undergoing pancreaticoduo‐denectomy: a cross‐sectional study (P12–025‐19). Medical Nutrition, 3(1), 8.

[fsn32310-bib-0014] Chiu, T.‐Y. , Liao, S.‐C. , Lee, W.‐C. , Lee, P.‐S. , Ng, H.‐Y. , Chien, Y.‐S. , & Lee, C.‐T. (2015). Gelsolin and adipokines are associated with protein‐energy wasting in hemodialysis patients. Artificial Organs, 39(2), 150–155. 25039281 10.1111/aor.12342

[fsn32310-bib-0015] Choi, H. Y. , Lee, J. E. , Han, S. H. , Yoo, T. H. , Kim, B. S. , Park, H. C. , Kang, S. W. , Choi, K. H. , Ha, S. K. , Lee, H. Y. , & Han, D. S. (2010). Association of inflammation and protein‐energy wasting with endothelial dysfunction in peritoneal dialysis patients. Nephrology, Dialysis, Transplantation, 25(4), 1266–1271. 10.1093/ndt/gfp598 19926717

[fsn32310-bib-0016] Dittmar, M. , Reber, H. , & Kahaly, G. J. (2015). Bioimpedance phase angle indicates catabolism in type 2 diabetes. Diabetic Medicine, 32(9), 1177–1185. 25661454 10.1111/dme.12710

[fsn32310-bib-0017] Duan, J. , Wang, C. , Liu, D. , Qiao, Y. , Pan, S. , Jiang, D. , Zhao, Z. , Liang, L. , Tian, F. , Yu, P. , Zhang, Y. U. , Zhao, H. , & Liu, Z. (2019). Prevalence and risk factors of chronic kidney disease and diabetic kidney disease in Chinese rural residents: A cross‐sectional survey. Scientific Reports, 9(1), 10408. 31320683 10.1038/s41598-019-46857-7 PMC6639314

[fsn32310-bib-0018] Gracia‐Iguacel, C. , González‐Parra, E. , Mahillo, I. , & Ortiz, A. (2019). Criteria for classification of protein‐energy wasting in dialysis patients: impact on prevalence. British Journal of Nutrition, 121(11), 1271–1278. 10.1017/S0007114519000400 31084673

[fsn32310-bib-0019] Hara, H. , Nakamura, Y. , Hatano, M. , Iwashita, T. , Shimizu, T. , Ogawa, T. , Kanozawa, K. , & Hasegawa, H. (2018). Protein energy wasting and sarcopenia in dialysis patients. Contributions to Nephrology, 196, 243–249. 30041234 10.1159/000485729

[fsn32310-bib-0020] Hasan, M. , Sutradhar, I. , Gupta, R. D. , & Sarker, M. (2018). Prevalence of chronic kidney disease in South Asia: A systematic review. BMC Nephrology, 19(1), 291. 30352554 10.1186/s12882-018-1072-5 PMC6199753

[fsn32310-bib-0021] Herrera Valdés, R. , Almaguer López, M. , Chipi Cabrera, J. A. , Pérez‐Oliva Díaz, J. F. , Landrove Rodríguez, O. , & Mármol Sóñora, A. (2020). Prevalence and incidence of chronic kidney disease in Cuba. Clinical Nephrology. 93, 68–71. 31549629 10.5414/CNP92S111

[fsn32310-bib-0022] Hill, N. R. , Fatoba, S. T. , Oke, J. L. , Hirst, J. A. , O’Callaghan, C. A. , Lasserson, D. S. , & Hobbs, F. D. R. (2016). Global prevalence of chronic kidney disease – A systematic review and meta‐analysis. PLoS One, 11(7), e0158765. 27383068 10.1371/journal.pone.0158765 PMC4934905

[fsn32310-bib-0023] Ishii, H. , Takahashi, H. , Ito, Y. , Aoyama, T. , Kamoi, D. , Sakakibara, T. , Umemoto, N. , Kumada, Y. , Suzuki, S. , & Murohara, T. (2017). The association of ankle brachial index, protein‐energy wasting, and inflammation status with cardiovascular mortality in patients on chronic hemodialysis. Nutrients, 9(4), 416. 10.3390/nu9040416 PMC5409755 28430145

[fsn32310-bib-0024] Karavetian, M. , Salhab, N. , Rizk, R. , & Poulia, K. A. (2019). Malnutrition‐inflammation score VS phase angle in the era of GLIM criteria: A cross‐sectional study among hemodialysis patients in UAE. Nutrients, 11(11), 2771. 10.3390/nu11112771 PMC6893836 31739568

[fsn32310-bib-0025] Kelly, L. , Matsumoto, C.‐L. , Schreiber, Y. , Gordon, J. , Willms, H. , Olivier, C. , Madden, S. , Hopko, J. , & Tobe, S. W. (2019). Prevalence of chronic kidney disease and cardiovascular comorbidities in adults in First Nations communities in northwest Ontario: A retrospective observational study. CMAJ Open, 7(3), E568–E572. 10.9778/cmajo.20190040 PMC6768774 31501170

[fsn32310-bib-0026] Krishnamoorthy, V. , Sunder, S. , Mahapatra, H. S. , Verma, H. , Sharma, N. , Jayaraman, R. , Sathi, S. , Khanna, S. , & Mohamed, A. (2015). Evaluation of protein‐energy wasting and inflammation on patients undergoing continuous ambulatory peritoneal dialysis and its correlations. Nephro‐Urology Monthly, 7(6), e33143. 26866011 10.5812/numonthly.33143 PMC4744638

[fsn32310-bib-0027] Leal Escobar, G. , Osuna Padilla, I. A. , Cano Escobar, K. B. , Moguel González, B. , Pérez Grovas, H. A. , & Ruiz Ubaldo S. (2019). Phase angle and mid arm circumference as predictors of protein energy wasting in renal replacement therapy patients. Nutricion Hospitalaria, 36(3), 633–639. 31192685 10.20960/nh.2463

[fsn32310-bib-0028] Lee, S. W. , Kim, Y.‐S. , Kim, Y. H. , Chung, W. , Park, S. K. , Choi, K. H. , Ahn, C. , & Oh, K.‐H. (2019). Dietary protein intake, protein energy wasting, and the progression of chronic kidney disease: Analysis from the KNOW‐CKD study. Nutrients, 11(1), 121. 10.3390/nu11010121 PMC6356719 30626166

[fsn32310-bib-0029] Lee, Y. , Kwon, O. , Shin, C. S. , & Lee, S. M. (2015). Use of bioelectrical impedance analysis for the assessment of nutritional status in critically ill patients. Clinical Nutrition Research, 4(1), 32–40. 25713790 10.7762/cnr.2015.4.1.32 PMC4337921

[fsn32310-bib-0030] Lv, J. C. , & Zhang, L. X. (2019). Prevalence and disease burden of chronic kidney disease. Advances in Experimental Medicine and Biology, 1165, 3–15. 31399958 10.1007/978-981-13-8871-2_1

[fsn32310-bib-0031] Monzani, A. , Perrone, M. , Prodam, F. , Moia, S. , Genoni, G. , Testa, S. , Paglialonga, F. , Rapa, A. , Bona, G. , Montini, G. , & Edefonti, A. (2018). Unacylated ghrelin and obestatin: promising biomarkers of protein energy wasting in children with chronic kidney disease. Pediatric Nephrology, 33(4), 661–672. 29150712 10.1007/s00467-017-3840-z

[fsn32310-bib-0032] Namuyimbwa, L. , Atuheire, C. , Okullo, J. , & Kalyesubula, R. (2018). Prevalence and associated factors of protein‐ energy wasting among patients with chronic kidney disease at Mulago hospital, Kampala‐Uganda: a cross‐sectional study. BMC Nephrology, 19(1), 139. 29902980 10.1186/s12882-018-0920-7 PMC6003131

[fsn32310-bib-0033] Norman, K. , Stobäus, N. , Pirlich, M. , & Bosy‐Westphal, A. (2012). Bioelectrical phase angle and impedance vector analysis–clinical relevance and applicability of impedance parameters. Clinical Nutrition, 31(6), 854–861. 22698802 10.1016/j.clnu.2012.05.008

[fsn32310-bib-0034] Panorchan, K. , & Davenport, A. (2017). Increase in extracellular hydration status after initiating peritoneal dialysis electively. Peritoneal Dialysis International: Journal of the International Society for Peritoneal Dialysis, 37(3), 338–340. 28512162 10.3747/pdi.2016.00213

[fsn32310-bib-0035] Player, E. L. , Morris, P. , Thomas, T. , Chan, W. Y. , Vyas, R. , Dutton, J. , Tang, J. , Alexandre, L. , & Forbes, A. (2019). Bioelectrical impedance analysis (BIA)‐derived phase angle (PA) is a practical aid to nutritional assessment in hospital in‐patients. Clinical Nutrition, 38(4), 1700–1706. 30170780 10.1016/j.clnu.2018.08.003

[fsn32310-bib-0036] Powrózek, T. , Brzozowska, A. , Mazurek, M. , Mlak, R. , Sobieszek, G. , & Małecka‐Massalska, T. (2019). Combined analysis of miRNA‐181a with phase angle derived from bioelectrical impedance predicts radiotherapy‐induced changes in body composition and survival of male patients with head and neck cancer. Head and Neck, 41(9), 3247–3257. 31165544 10.1002/hed.25830

[fsn32310-bib-0037] Ranasinghe, A. V. , Kumara, G. W. G. P. , Karunarathna, R. H. , De Silva, A. P. , Sachintani, K. G. D. , Gunawardena, J. M. C. , Kumari, S. K. C. R. , Sarjana, M. S. F. , Chandraguptha, J. S. , & De Silva, M. V. C. (2019). The incidence, prevalence and trends of chronic kidney disease and chronic kidney disease of uncertain aetiology (CKDu) in the North Central Province of Sri Lanka: an analysis of 30,566 patients. BMC Nephrology, 20(1), 338. 31462219 10.1186/s12882-019-1501-0 PMC6714078

[fsn32310-bib-0038] Sasaki, N. , & Al, E. (2008). The optimal ratio of extracellular water to total body water (ECW/TBW) determined by bioelectrical impedance analysis (BIA) for setting dry weight in hemodialysis patients. Journals of Hemodialysis, 41(10), 723–730.

[fsn32310-bib-0039] Shin, J.‐H. , Kim, C. R. , Park, K. H. , Hwang, J. H. , & Kim, S. H. (2017). Predicting clinical outcomes using phase angle as assessed by bioelectrical impedance analysis in maintenance hemodialysis patients. Nutrition, 41, 7–13. 28760431 10.1016/j.nut.2017.02.013

[fsn32310-bib-0040] Singh, N. , Fallahzadeh, M. K. , & Abreo, K. (2015). Effect of increased dietary protein intake on the 24‐hour urine creatinine clearance and eligibility of living kidney donor candidates. Experimental and Clinical Transplantation: Official Journal of the Middle East Society for Organ Transplantation, 13(6), 609–610. 26643680

[fsn32310-bib-0041] Tan, R.‐S. , Liang, D.‐H. , Liu, Y. , Zhong, X.‐S. , Zhang, D.‐S. , & Ma, J. (2019). Bioelectrical impedance analysis‐derived phase angle predicts protein‐energy wasting in maintenance hemodialysis patients. Journal of Renal Nutrition, 29(4), 295–301. 30446269 10.1053/j.jrn.2018.09.001

[fsn32310-bib-0042] Vermeulen, K. M. , Lopes, M. M. G. D. , Alves, C. X. , Brito, N. J. N. , Almeida, M. D. G. , Leite‐Lais, L. , Vale, S. H. L. , & Brandão‐Neto, J. (2019). Bioelectrical impedance vector analysis and phase angle on different oral zinc supplementation in eutrophic children: Randomized triple‐blind study. Nutrients, 11(6), 1215. 10.3390/nu11061215 PMC6627540 31142044

[fsn32310-bib-0043] Vermeulen, K. M. , Lopes, M. M. G. D. , Grilo, E. C. , Alves, C. X. , Machado, R. J. A. , Lais, L. L. , Brandão‐Neto, J. , & Vale, S. H. L. (2019). Bioelectrical impedance vector analysis and phase angle in boys with Duchenne muscular dystrophy. Food & Nutrition Research, 63, 1–9. 10.29219/fnr.v63.1615 PMC6458958 31007651

[fsn32310-bib-0044] Windahl, K. , Faxén Irving, G. , Almquist, T. , Lidén, M. K. , van de Luijtgaarden, M. , Chesnaye, N. C. , Voskamp, P. , Stenvinkel, P. , Klinger, M. , Szymczak, M. , Torino, C. , Postorini, M. , Drechsler, C. , Caskey, F. J. , Wanner, C. , Dekker, F. W. , Jager, K. J. , & Evans, M. (2018). Prevalence and risk of protein‐energy wasting assessed by subjective global assessment in older adults with advanced chronic kidney disease: results from the EQUAL study. Journal of Renal Nutrition, 28(3), 165–174. 29459026 10.1053/j.jrn.2017.11.002

[fsn32310-bib-0045] Yamada, M. , Kimura, Y. , Ishiyama, D. , Nishio, N. , Otobe, Y. , Tanaka, T. , Ohji, S. , Koyama, S. , Sato, A. , Suzuki, M. , Ogawa, H. , Ichikawa, T. , Ito, D. , & Arai, H. (2018). Phase angle is a useful indicator for muscle function in older adults. The Journal of Nutrition, Health & Aging, 23(3), 251–255.10.1007/s12603-018-1151-030820513

[fsn32310-bib-0046] Yoshida, M. , Asagiri, K. , Fukahori, S. , Tanaka, Y. , Hashizume, N. , Ishii, S. , Saikusa, N. , Higashidate, N. , Masui, D. , Komatsuzaki, N. , Nakahara, H. , Yagi, M. , & Yamashita, Y. (2017). The utility of a phase angle analysis in patients with severe motor and intellectual disabilities. Brain and Development, 39(7), 557–563. 28365067 10.1016/j.braindev.2017.03.003

[fsn32310-bib-0047] Yuan, J. , Watanabe, M. , Suliman, M. , Qureshi, A. R. , Axelsson, J. , Barany, P. , Heimburger, O. , Stenvinkel, P. , & Lindholm, B. (2015). Serum hepatocyte growth factor is associated with truncal fat mass and increased mortality in chronic kidney disease stage 5 patients with protein‐energy wasting. Nephrology, Dialysis, Transplantation, 30(2), 274–282. 10.1093/ndt/gft265 23975839

[fsn32310-bib-0048] Zhou, M. , Wang, H. , Zeng, X. , Yin, P. , Zhu, J. , Chen, W. , Li, X. , Wang, L. , Wang, L. , Liu, Y. , Liu, J. , Zhang, M. , Qi, J. , Yu, S. , Afshin, A. , Gakidou, E. , Glenn, S. , Krish, V. S. , Miller‐Petrie, M. K. , … Liang, X. (2019). Mortality, morbidity, and risk factors in China and its provinces, 1990–2017: A systematic analysis for the Global Burden of Disease Study 2017. The Lancet, 394(10204), 1145–1158. 10.1016/S0140-6736(19)30427-1 PMC6891889 31248666

